# An Approach to Heterologous Expression of Membrane Proteins. The Case of Bacteriorhodopsin

**DOI:** 10.1371/journal.pone.0128390

**Published:** 2015-06-05

**Authors:** Dmitry Bratanov, Taras Balandin, Ekaterina Round, Vitaly Shevchenko, Ivan Gushchin, Vitaly Polovinkin, Valentin Borshchevskiy, Valentin Gordeliy

**Affiliations:** 1 Institute of Complex Systems (ICS), ICS-6: Structural Biochemistry, Research Centre Jülich, Jülich, Germany; 2 Institute of Crystallography, University of Aachen (RWTH), Jägerstrasse 17–19, Aachen, Germany; 3 Univ. Grenoble Alpes, IBS, Grenoble, France; 4 CNRS, IBS, Grenoble, France; 5 CEA, IBS, Grenoble, France; 6 Research-Educational Centre “Bionanophysics”, Moscow Institute of Physics and Technology, Dolgoprudniy, Russia; University of Bern, SWITZERLAND

## Abstract

Heterologous overexpression of functional membrane proteins is a major bottleneck of structural biology. Bacteriorhodopsin from *Halobium salinarum* (bR) is a striking example of the difficulties in membrane protein overexpression. We suggest a general approach with a finite number of steps which allows one to localize the underlying problem of poor expression of a membrane protein using bR as an example. Our approach is based on constructing chimeric proteins comprising parts of a protein of interest and complementary parts of a homologous protein demonstrating advantageous expression. This complementary protein approach allowed us to increase bR expression by two orders of magnitude through the introduction of two silent mutations into bR coding DNA. For the first time the high quality crystals of bR expressed in *E*. *Coli* were obtained using the produced protein. The crystals obtained with *in meso* nanovolume crystallization diffracted to 1.67 Å.

## Introduction

The membrane protein (MP) genes comprise about one third of the human genes encoding proteins. In spite of the recent success with GPCRs [1, 2], membrane proteins (MPs) still only comprise about 1% of total structures in the PDB [3, 4]. One of the bottlenecks in structural biology for MPs is the difficulty of production of pure and functional MPs, especially of human origin. However even bacterial and archea MPs often do not express heterologously. One of the striking examples is the expression of bacteriorhodopsin from *Halobium salinarum* (bR) in *E*. *coli*.

Bacteriorhodopsin is a light-driven pump that provides proton vectorial transport across the cell membrane of the archaea [5, 6]. It consists of 7 transmembrane α-helices (7TM) with the retinal molecule bound covalently to the Lys 216 residue [7] and belongs to the 7TM protein superfamily. As part of one of the simplest energy production mechanisms in the cell, bR is of great interest for bioenergetics. Due to the high level of homologous expression, combined with the ease of purification from the natural source [8] and high thermal and chemical stability [9] this protein has become the most studied MP. BR is a widely used model for expression, folding, crystallization of MPs and X-ray crystallography. Highly ordered 3D crystals of bR [10, 11] significantly improved understanding of the molecular mechanism of MP function and of vectorial proton transport in particular [12]. This protein and its mutants are in high demand in studies regarding their applications in bioelectronics, optics, and optogenetics [13–15]. Unfortunately, homologous production of bR mutants and many other light-driven proteins is laborous, time- and resource-consuming or is simply not possible at all.

The bR functional expression in *E*. *coli* would be ideal as it is the most simple, robust, and inexpensive system [16]. However, for almost 30 years continued efforts towards this goal have been unsuccessful. The first studies showed a low level of wild type bR expression in *E*. *coli* due to severe degradation of the newly synthesized protein [17, 18]. Application of exogenous *N*-terminal tags has been used to stabilize the protein and prevent its degradation thereby increasing the bR yield to 17 mg of protein per litre of culture [18, 19]. Further improvements in yield were from the use of fusion proteins giving expression of 100–200 mg/l [20–22]. However, despite the high yield the above mentioned systems did not result in functional bR expression requiring refolding of the purified protein. Such behavior of bR is surprising, as a number of closely related retinal proteins were expressed in a functional form in *E*. *coli*, namely halorhodopsin and sensory rhodopsin II from *Natronomonas pharaonis* (hR and SRII, respectively) [23, 24], rhodopsin from *Exiguobacterium sibiricum* (ESR) [25], deltarhodopsin from *Haloterrigena turkmenica* [26], and bacteriorhodopsin from *Haloarcula marismortui* [27].

**Fig 1:**
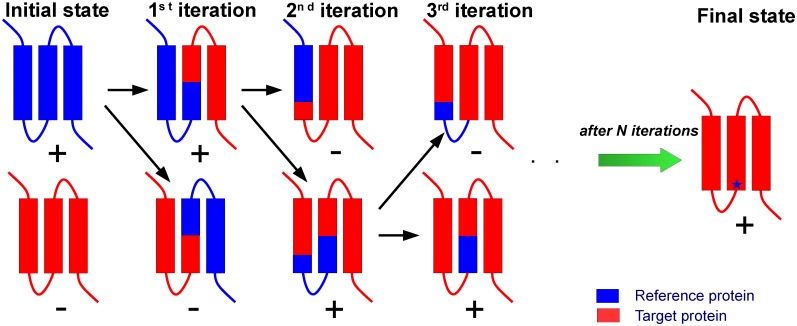
Representation of the complementary protein approach. Two homologous MPs reference protein (blue) and target protein (red) which have high and low (or zero) expression in a selected organism respectively. One can construct chimeric proteins (1^st^ iteration) comprising parts of the target protein and the reference protein. Analyzing expression yields of the chimeric proteins one can determine which part of the target protein is responsible for a heterologous expression failure. In the next step, one can divide this part of the protein and construct the next chimeric proteins (2^nd^ iteration). Acting in the same manner one can finally localize the problem of the lack of high yield expression of the target protein (showed as blue star) in a limited number of steps. One can estimate the required number of genetic constructs as 2∙log_2_N instead of 2^N^ point mutations in the case of a random search for the problematic parts of a protein responsible for the failure of the expression, where N is the number of amino acids in the target protein.

This raises the questions why are the other 7TM retinal proteins readily overexpressed in *E*. *coli* and why is bR not? What is the difference between bR and SRII that allows functional expression of SRII in *E*. *coli*? We addressed these questions by applying the complementary protein approach (CPA, shown in Fig 1). Chimeric proteins were constructed which comprised a part of bR and a complementary part of a reference protein SRII. SRII was chosen as this retinal protein was expressed functionally in *E*. *coli* [23, 24] and has high sequence identity to bR (27.49%) plus the high resolution 3D crystal structure is available [28, 29]. This approach allowed us to localize quickly the reason for the lack of bR expression in *E*. *coli* and we suggest that it may have a general application.

## Results

### Identification of the part of bacteriorhodopsin amino acid sequence responsible for failure of its expression in E. coli and strong influence of positively charged Arg7 at N-terminus

**Fig 2:**
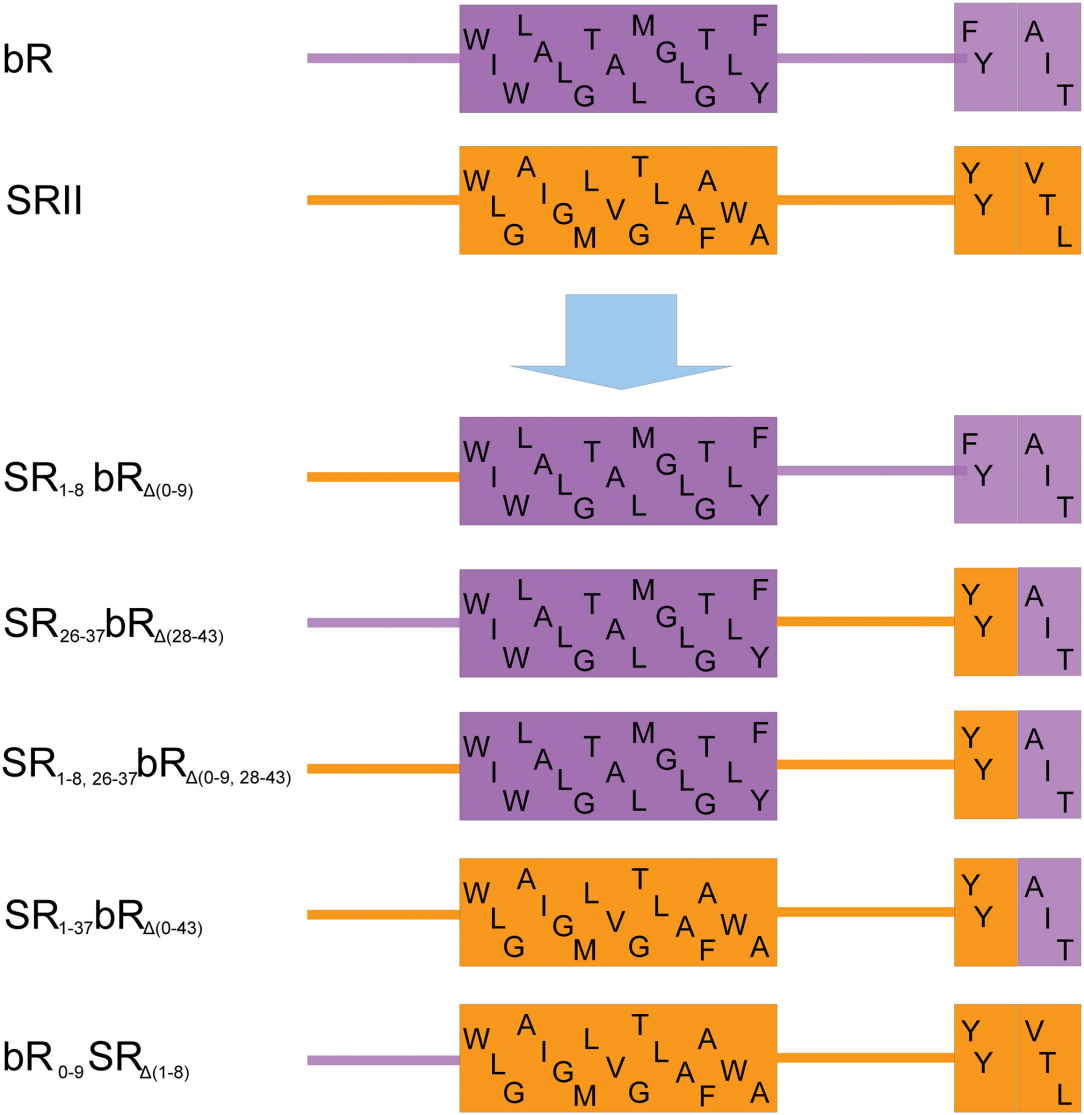
Design of chimeric proteins between bR and SRII. *N*-terminus, first α-helix and the beginning of the second α-helix of bR (purple) and SRII (orange) with the corresponding amino acid sequence are shown. Subscript in the name of the construct indicates amino acids of bR that were replaced by the counterparts from SRII and vice versa.

**Fig 3:**
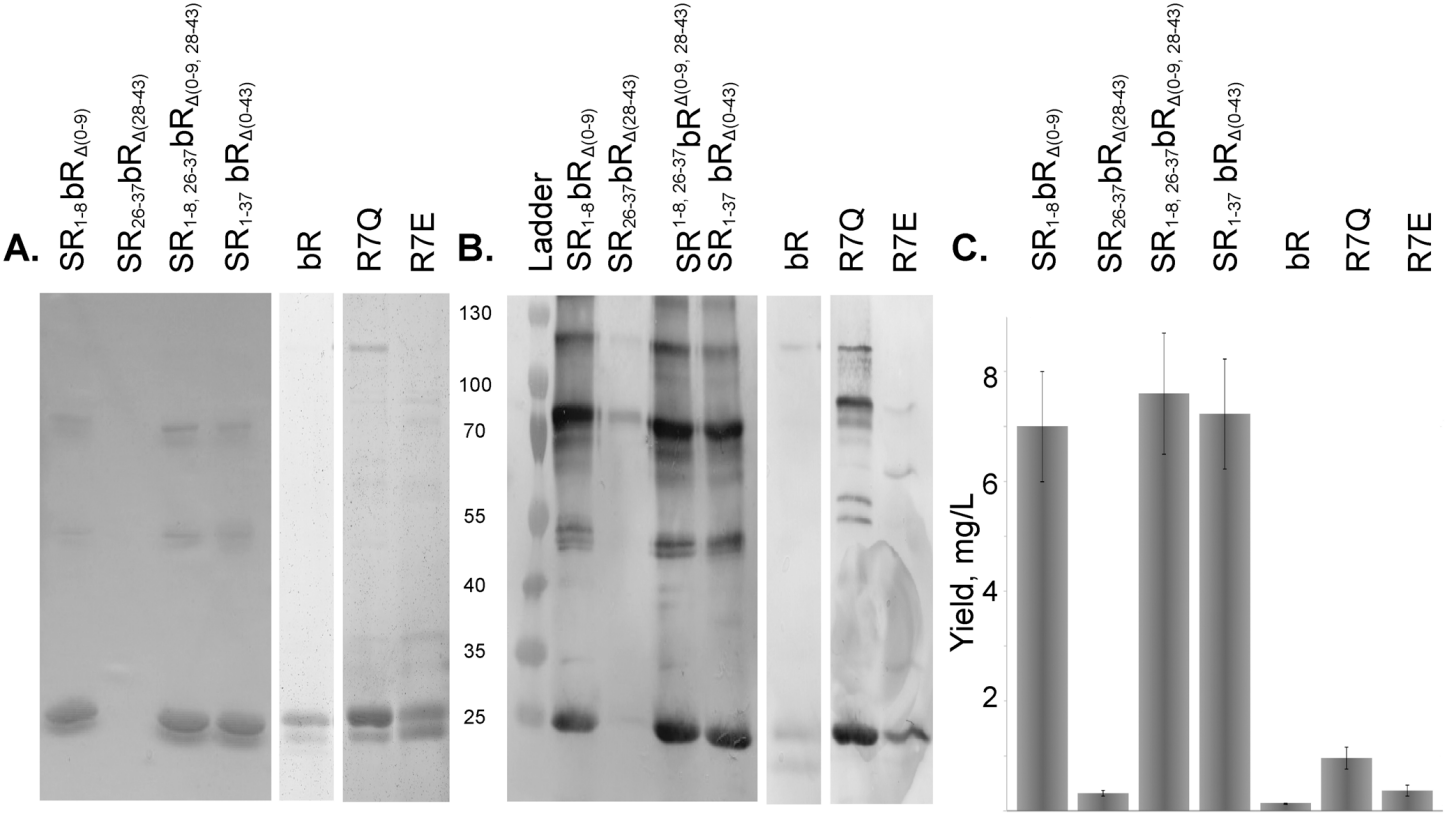
SDS-PAGE analysis of chimeric proteins, point mutants purified by metal affinity chromatography under denaturing conditions. A. coomassie-stained gels. B. corresponding anti-*His*-tag antibody stained imunoblots. The amount of the protein loaded was normalized to the total amount of cell culture. C. The yields of the chimeric proteins and point mutants as quantified by the BCA protein assay, band density on SDS-PAGE and Western blotting. Chimeric proteins containing extracellular *N*-terminus of SRII have shown a much better yield.

We constructed chimeric proteins by combining complementary parts of bR and SRII (Fig 2). The names of chimeric proteins indicate the complementary residues which have been replaced in subscript. For example, we replaced the initial 43 amino acids of bR by the corresponding 36 amino acids of SRII to make chimeric protein SR_1-37_bR_Δ(0–43)_. To elucidate the influence of extracellular, transmembrane and cytoplasmic regions independently we constructed SR_1-8_bR_Δ(0–9)_, SR_26-37_bR_Δ(28–43)_, SR_1-8,26-37_bR_Δ(0–9,28–43)_, respectively (Fig 2). The proteins were expressed in *E*. *coli*, solubilized and purified from membranes isolated by ultracentrifugation using metal affinity chromatography under denaturing conditions. Identity of the purified proteins was confirmed by Western blotting with anti-*His*-tag antibodies. Replacement of the first 9 amino acids on the *N*-terminus of bR by the counterparts from SRII significantly improves the expression of the native bR gene (Fig 3). The yields of SR_1-8_bR_Δ(0–9)_ and the native bR were quantified by a BCA protein assay as 7.0±1.0 and 0.14±0.04 mg/l, respectively. The yield of chimeric protein is comparable with that of the native bR in *Halobium salinarum* (30 mg/litre of culture, [8]) and sufficient for structural studies and other purposes. Both chimeric proteins SR_1-8_bR_Δ(0–9)_ and SR_1-37_bR_Δ(0–43)_ purified under non-denaturing conditions were not functional, but were renaturated using an established procedure of bR retinalization in DMPC/CHAPS micelles [30]. The reverse construct bR_0-9_SR_Δ(1–8)_ containing the first 10 amino acids of bR instead of their counterparts on the *N*-terminus of SRII (Fig 2) yielded 6.0±1.8 mg/l comparing to 18.1±2.8 mg/l for SRII.

The point mutants R7Q and R7E eliminating the only positive charge on extracellular *N*-terminus that do not satisfy “positive inside” rule were expressed and purified following the denaturing protocol with the yields of 0.96±0.20 mg and 0.37±0.10 mg of protein per litre of culture, respectively (Fig 3), which are correspondingly 7 and 3 times higher than that for the native bR but still 7 times lower than for a chimeric gene SR_1-8_bR_Δ(0–9)_.

### Optimization of mRNA remarkably increases the yield of wild type bacteriorhodopsin

**Fig 4:**
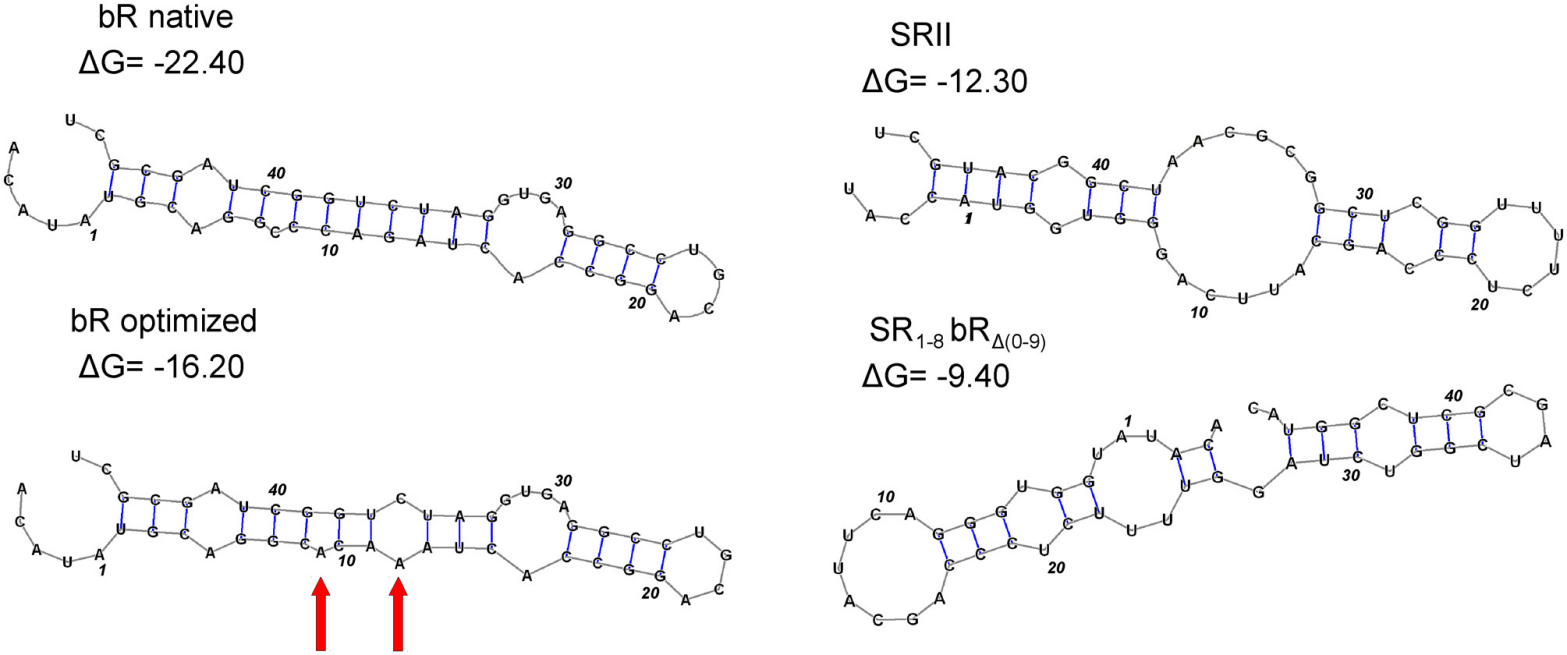
The mRNA shapes of the native bR, SRII, chimeric protein SR_1-8_bR_Δ(0–9)_, and optimized bR gene. The region (-4, +47) with numbering starting from ATG start codon is shown. This region corresponds to amino acids from Met0 to Leu15. Free energies presented are expressed in *kcal/mol*. The values of mRNA free energy for SRII and SR_1-8_bR_Δ(0–9)_ are considerably smaller than those of the native bR gene. The mRNA structure optimization of bR by silent mutations increased the free energy of the region (-4, +47) by 6.2 *kcal/mol*. The red arrows indicate the mutated nucleotides.

Using the mRNA structure prediction software *mRNAshapes* [31] we found a putative stem structure at the start of the bR gene (Fig 4). As the stem stretches beyond nucleotide 37, we had to expand the region under consideration to (-4, +47) comparing to the previous study [32]. This region corresponds to amino acids from Met0 to Leu15. The free folding energy of mRNA for the (-4, +47) region of bR is predicted to be -22.4 *kcal/mol* (Fig 4), while the corresponding value for SRII, ESR, and hR genes are -12.3, -5.2, and -13.1 *kcal/mol* respectively, indicating the decreased stability of the mRNA structures at the 5'-terminus near the ribosome binding site. The change of the first 9 codons of bR to the counterparts of SRII considerably weakened the interactions between the 6–15 and 33–41 regions of this stem and increased the free energy by 13.0 *kcal/mol*. Conversely, the folding energy of bR_0-9_SR_Δ(1–8)_ is -15.5 *kcal/mol* which accounts for the lower yield of this chimera. Since the expression levels of the native bR, SRII, SR_1-8_bR_Δ(0–9)_, and bR_0-9_SR_Δ(1–8)_ correlated with the stability of the (-4, +47) region of mRNA structure, we introduced into the wild type bR gene two silent mutations (C9A, G12A corresponding to amino acids Ala2 and Gln3) that increase the free energy by 6.2 *kcal/mol* and significantly reduce the stem stability (Fig 4). The optimized bR gene was expressed in *E*. *coli* and purified following the denaturing protocol. The yield of the protein in case of the optimized gene was quantified as 9.2±3.5 mg per litre of culture, when measured by BCA protein assay, being essentially the same as that of SR_1-8_bR_Δ(0–9)_ (*p* = 0.74) in contrast to the low yield of native gene (Fig 3).

### Purification and characterization of functional wild-type bacteriorhodopsin under non-denaturing conditions

When we utilized a mild detergent for solubilization of *E*. *coli* membranes and the non-denaturing protocol for protein purification, bR expressed from the optimized gene retained its purple color. The first step of purification by metal affinity chromatography led to mixed functional and nonfunctional bR preparations. The total yield of the protein after affinity purification was 7.6±2.9 mg per litre of culture, as measured by BCA protein assay. Sample homogeneity was confirmed by coomassie-stained SDS-PAGE.

As solubilized bR is unstable at alkaline pH and imidazole is harmful for the protein, we removed imidazole and adjusted pH to 6.0 by dialysis. During the pH exchange the protein heavily precipitated. The pellet was not colorled and constituted of bR according to SDS-PAGE analysis suggesting aggregation of the misfolded protein. The pellet was discarded leaving the functional bR in the supernatant, however UV-Vis spectroscopy showed that the sample still contained aggregates and protein contaminants.

**Fig 5:**
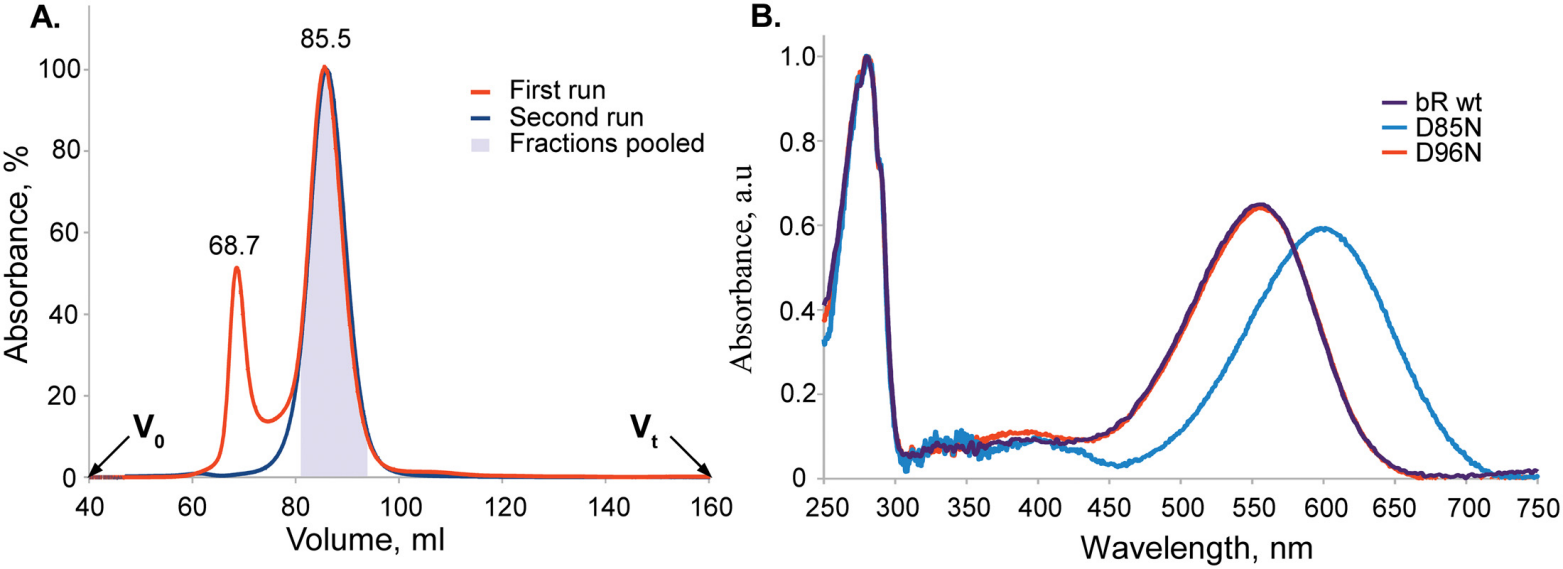
Characterization of bR expressed in *E*. *coli*. A. Elution profile of the wild type bR on Sephacryl S200HR. The initial run is shown in red and the repeat run (after storage) in blue. The peak at 68.68 ml in the first run corresponds to the aggregated colorless protein. The second peak at 85.54 ml is the functional target protein. The fractions corresponding to the second peak were colored and those in the grey box were pooled. The repeat run after 5 days does not show any significant amount of aggregates. B. UV-Vis absorption spectra of the samples of the wild type bR and D85N and D96N mutants normalized by absorbance at 280 nm. Proteins were solubilized in DDM and purified using non-denaturing protocol. The spectrum of the wild type bR exhibited the retinal absorption peak at 555.5±1.0 nm with a peak ratio A_280_/A_λret_ of 1.5 which corresponds to the highest purity of bR.

Further purification of bR utilised size-exclusion chromatography (SEC) yielding two distinct peaks at 69 ml and 86 ml corresponding to bR aggregates and functional bR respectively (Fig 5). The colored fractions from the latter peak were pooled, mixed and concentrated. The UV-Vis absorbance spectrum exhibited the retinal absorption peak at 555.5±1.0 nm. A peak ratio at A_280_/A_λret_ of 1.5 was achieved (Fig 5), demonstrating that the purity of the protein is consistent with that of bR solubilized from native purple membranes of *H*. *salinarum*. Storage stability of the protein was analysed 5 days after purification by repeat SEC on the same column. Bacteriorhodopsin eluted as a single and symmetric (asymmetry index 1.05) peak indicating size homogeneity of the final product. This confirmed that the aggregates were removed completely from the samples and the purified protein had no tendency to denature or form aggregates during storage. Prolonged storage indicated that the protein half-life at 4°C exceeds 120 days. The final yield of the purified functional wild type bR expressed in *E*. *coli* was 2.4±1.3 mg of the protein per litre of culture corresponding to 15–35% of the total synthesized bR.

### Expression, functional purification, and characterization of D85N and D96N mutants of bacteriorhodopsin

Using the mRNA-optimized bR gene we have introduced mutations D85N and D96N and utilizing the non-denaturing purification protocol we readily obtained functional mutant proteins with the final yields of 3.8 and 8.8 mg per litre of culture, respectively. The mutant proteins had essentially the same gel-filtration elution profiles as the wild-type bR. D96N UV-Vis spectrum was simular to the wild-type bR, while D85N mutant exhibited the characteristic maximum retinal absorbance for this protein at 598.0 nm (Fig 5).

### Crystallization of the purified proteins

**Fig 6:**
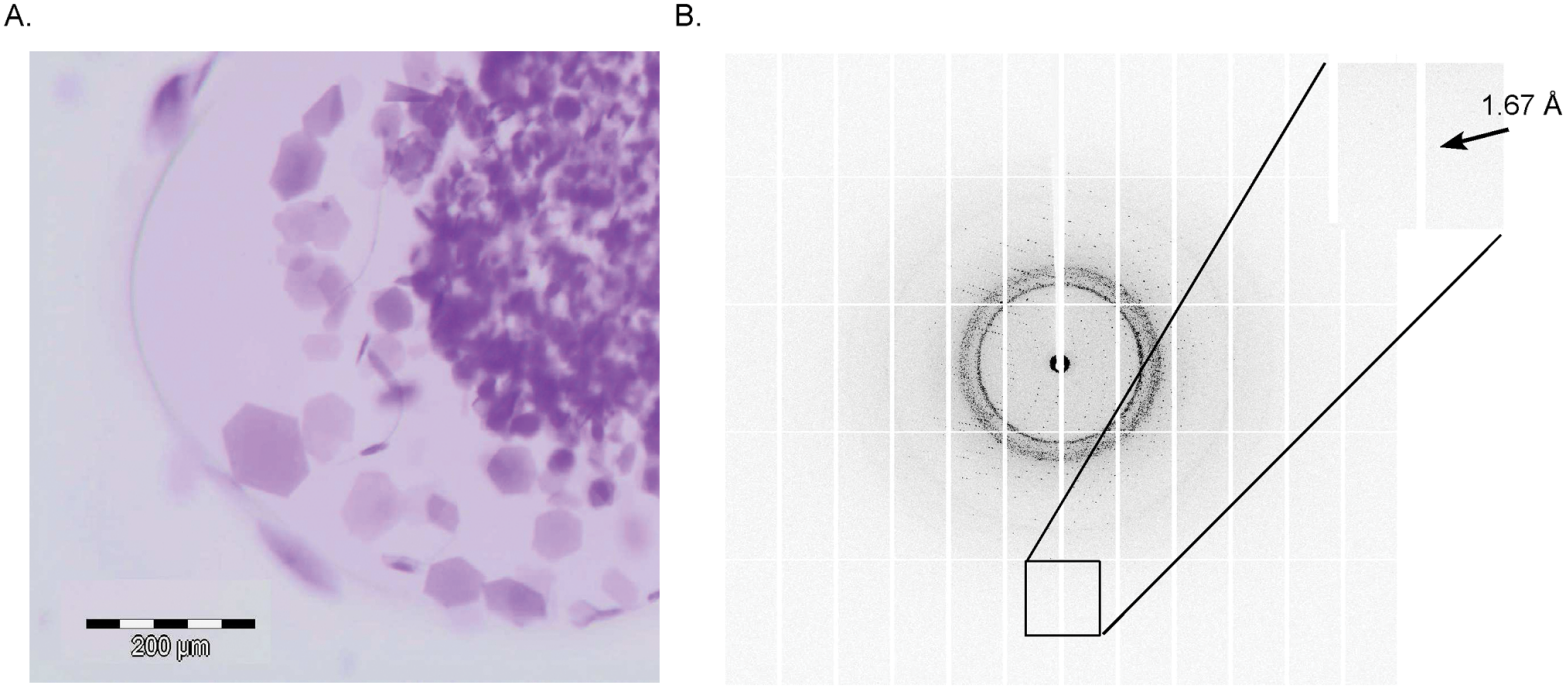
Crystals of bR D96N mutant. A. Crystals of bR D96N mutant expressed in *E*. *coli* were obtained using the *in meso* nanovolume crystallization approach. The largest crystals had the size of 120 μm. B. A crystal (without further optimization of crystallization conditions) showed diffraction to 1.67 Å (inset).

**Fig 7:**
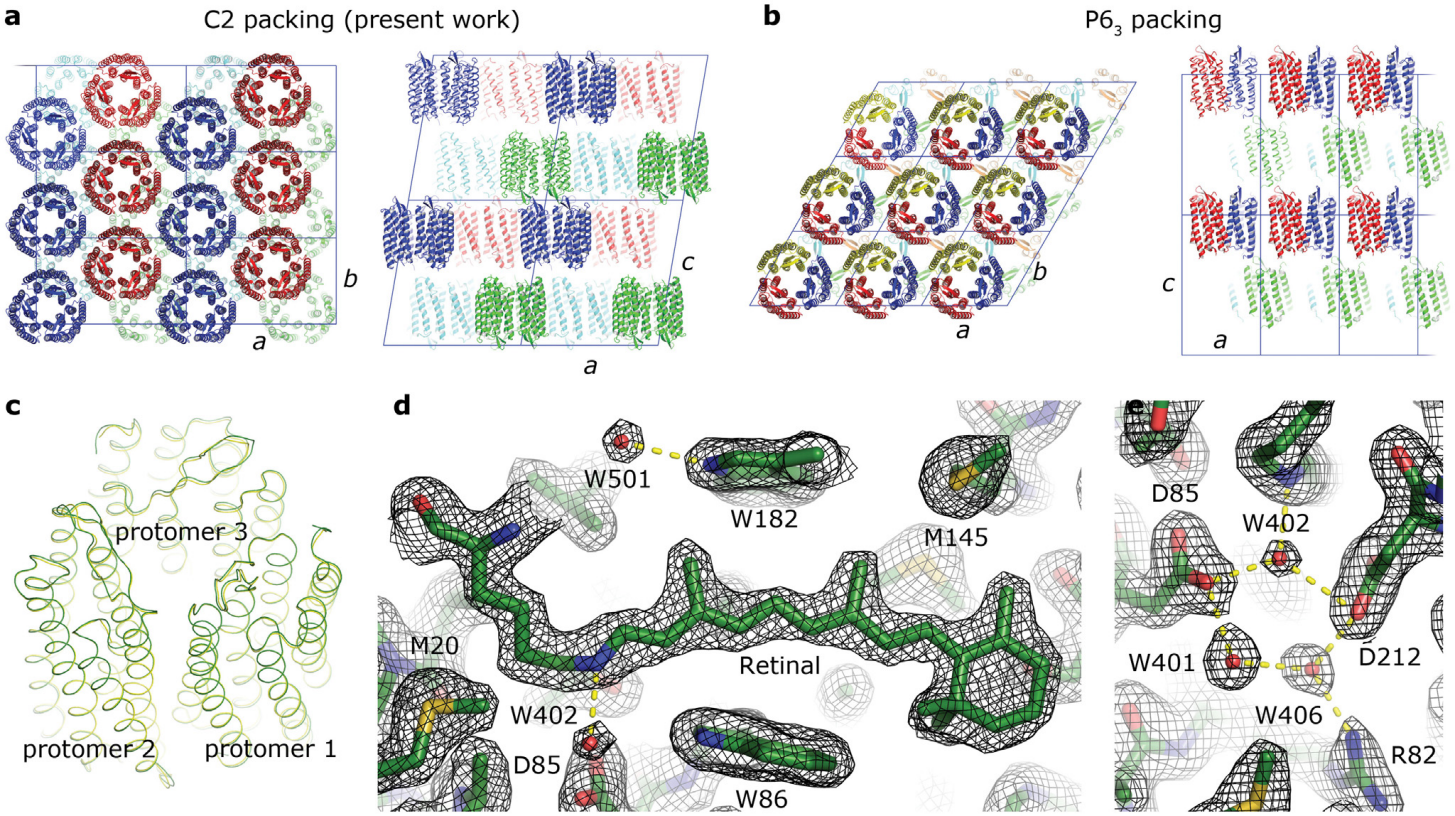
Crystallographic structure of *E*. *coli*-expressed bacteriorhodopsin. A. Packing of *E*. *coli*-expressed bacteriorhodopsin in the C2 crystals (three molecules per asymmetric unit). The inter-trimer distance is 60.76 Å. B. Packing of bacteriorhodopsin from purple membrane in the P6_3_ crystals (one molecules per asymmetric unit). The inter-trimer distance is 60.63 Å [11]. C Comparison of the trimer structure in the C2 (green) and P6_3_ (yellow) crystals. The structures are essentially identical with r.m.s.d. of the atom positions of less than 0.3 Å. D and E 2F_o_-F_c_ electron densities around the retinal D and its Schiff base E contoured at the level of 1.5 σ. Hydrogen bonds are shown in yellow.

With the wild type bR, D85N and D96N mutant proteins we set up nanovolume *in meso* crystallization trials [10, 33] which yielded crystals within 5–10 days for all proteins. The crystals of wild type protein and D96N mutant had a shape of thin hexagonal plates of 120 *μ*m (Fig 6). The single crystals obtained without optimization were tested at a synchrotron beam line and gave difraction up to 1.67 Å resolution (Fig 6). The structure was solved with a resolution of 1.9 Å (see Table 1 for Crystallographic data statistics) and deposited in the PDB with ID code of 4XXJ. The crystals belonged to the spacegroup C2 with three monomers in the asymmetric unit that form a trimer. Typically for *in meso* crystallization, the protein molecules form membrane-like layers, with purple membrane-like packing within the layer (Fig 7). Despite the absence of native *Halobacterium salinarum* lipids, the intertrimer distance is essentially identical to that of the crystals obtained with purple membrane-derived bacteriorhodopsin. The structure of the protein and its trimer is also identical to that of purple membrane-derived bacteriorhodopsin (Fig 7). Data reveal the essential features of the structure, including the retinal cofactor and the hydrogen bond-linked cluster of water molecules close to the Schiff base (Fig 7).

**Table 1:** Crystallographic data collection and refinement statistics.

Data collection	
Space group	C2
Cell dimensions	
*a*, *b*, *c* (Å)	105.84, 60.76, 113.36
α, β, γ (°)	90, 99.78, 90
Resolution (Å)	49.2–1.9 (1.95–1.9)*
*R*_merge_ (%)	7.1 (110.2)*
*I/σI*	13.7 (1.41)*
Completeness (%)	98.8 (98.3)*
Multiplicity	4.5 (4.5)*
**Refinement**	
Resolution	49.2–1.9 Å
No. reflections	56113 (2796**)
*R*_work_ / *R*_free_	18.35% / 21.39%
Number of atoms	
Protein	5306
Retinal	60
Lipid	248
Water	90
Average B-factor (Å^2^)	
Protein	34.4
Retinal	25.6
Lipid	51.2
Water	39.0
R.m.s. deviations	
Bond lengths	0.005 Å
Bond angles	0.9°

* Values in parentheses are for the highest-resolution shell.

** Number of reflections that are not used for refinement (free reflections)

## Discussion

**Fig 8:**
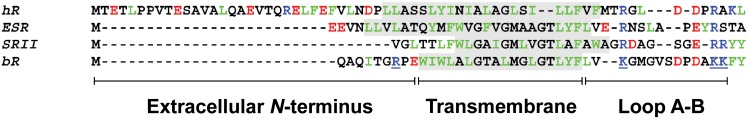
Sequence alignment of HR, ESR, SRII and bR amino acids of the helix A. Positively charged, negatively charged and hydrophobic amino acids are marked with blue, red and green colors respectively. Transmembrane domains of helix A as determined from the structure are marked with light grey. These specific features of bR, which may affect expression of the protein, were revealed in bR and are underlined.

In spite of the high level of sequence identity bR did not show functional expression in *E*. *coli* whereas to SRII, hR, and ESR did. To determine why this is the case we looked more coldly at the differences in the amino acid sequences (Fig 8). The “positive inside” rule states that MP topogenesis is controlled by positively charged amino acids [34] that keep the cytoplasmic parts of MP in the cytoplasm. This is due to the interactions with negatively charged headgroups of anionic lipids [35, 36] and these amino acids can therefore withstand both Sec-dependent and Sec-independent translocation against membrane potential. As observed *in vitro*, via the use of lysates and inner membrane vesicles (prepared from *E*. *coli*) bR is inserted co-translationally into the *E*. *coli* membrane in a Sec-dependent manner [37] proving the amino acid sequence of the first transmembrane helix and adjacent regions to be particularly important for the insertion of integral MPs into membrane.

The alignment of bR, SRII, ESR, and hR revealed two distinct features of bacteriorhodopsin in the region of the first transmembrane helix. First, the positively charged Arg7 on the extracellular *N*-terminus of bacteriorhodopsin deviates from the "positive inside" rule. Second, bacteriorhodopsin has a different positively charged amino acid pattern in the cytoplasmic loop A-B, where lysines substitute for the arginines in the vicinity of the hydrophobic-hydrophilic interface. Such a charge distribution on the membrane flanking regions of the first helix may compromise the folding and expression rate of bR. Thus, this preliminary considerations helped us to select the bR region that may contain parts of the protein problematic for *E*. *coli* expression. To investigate whether the charge distribution in the helix A region influences bR expression level in *E*. *coli* and the insertion of the protein into bacterial membrane we constructed several chimeric proteins by combining complementary parts of bR and SRII (Fig 2), as the latter is expressed in *E*. *coli* functionally with a high yield [23] and satisfies the "positive inside" rule (Fig 8).

There are already literature data on the construction of chimeric proteins between homologous proteins to find, for example, amino acid residues determining functional differences between these homologs (e.g. [38]). Moreover, on occasions when the chimeric proteins have a high expression yield or increased stability, they are expressed and even crystallized instead of their ancestors [39]. However, the systematic studies of the reason for the *E*. *coli* MP expression failure that involve the use of the chimeric proteins were lacking. Here, using bR as an example we show how the described approach can be employed for efficient MP production. A gain of over 50 times in the yield of bR expressed in *E*. *coli* was reached by the replacement of extracellular N-terminus of bR with the SRII amino acid sequence. It should be stressed that the part of the protein “responsible” for the failure of bR expression in *E*. *coli* was localized in 2 steps using 4 chimeric proteins. In general, CPA allows one to reduce the required number of genetic constructs from 2^N^ point mutations to 2∙log_2_N chimeric proteins, where N is the number of amino acids in the target protein.

Interestingly, the construct bR_0-9_SR_Δ(1–8)_ containing the first 10 amino acids of bR instead of their counterparts on the *N*-terminus of SRII (Fig 2) yielded 6.0±1.8 mg/l comparing to 18.1±2.8 mg/l for SRII. Spectral tuning of SRII by constructing chimeric proteins between SRII and bR [40] produced in small amount the functional chimera comprising A-C helices of bR and D-G helices of SRII expressed in *E*. *coli*. In the case of the bR_0-9_SR_Δ(1–8)_ construct under non-denaturing conditions a functional protein was not obtained. We hypothesize that the introduction of charged residues into the hydrophobic interface of helix A brakes its hydrophobic interactions with the other helices leading to the destabilization of the SRII structure and thus misfolding of the bR_0-9_SR_Δ(1–8)_ chimera. Therefore, the suppressing influence of the extracellular *N*-terminus of bR has been supported by deterioration of functional SRII expression in *E*. *coli* when the *N*-terminus of SRII was replaced with its counterpart from bR.

Since the low expression level of wild type bR in *E*. *coli* comparing to SR_1-8_bR_Δ(0–9)_ could be caused by the unfavorable mRNA structure of the bR gene near the ribosome binding site or the presence of positively charged Arg7 at the *N*-terminus side of the helix A of bR, we studied the influence of these two factors separately. First, we replaced Arg7 of the native bR gene by either neutral or negatively charged residue and constructed mutants R7Q and R7E respectively. The observed relationship between expression levels of native bR, R7Q and R7E mutants, and the SR_1-8_bR_Δ(0–9)_ chimera in *E*. *coli* (Fig 3) could be explained in part by the "positive inside" rule as the removal of the positive charge on the *N*-terminus of bR improves the yield of the protein. However, these experiments showed that it is not the only reason for the low expression level of bR in *E*. *coli*.

The stability of mRNA folding near the ribosome binding site is known to have a strong impact on the protein expression level [32]. It has been shown that the variation of the nucleotide sequence near the ribosome binding site significantly influences the expression level of bR in *E*. *coli*, but functional incorporation of the protein into membrane has not been achieved [18]. Using the mRNA structure prediction software *mRNAshapes* [31] we found a putative stem structure at the start of the bR gene. The optimized bR gene yielded essentially the same amount of protein as the chimeric proteins in contrast to the native gene as demonstrated by Fig 3. Thereby, we showed that destabilization of the putative stem structure in the 5'-terminus of mRNA leads to a significant increase in the expression level of the protein.

The optimization of mRNA has also significantly increased the bR expression rate in the previous study with the use of pJP plasmid [18]. Additionally, in the mRNA 5' untranslated region the hairpin structure was found. It encloses the *lac* operator and hence impairs mRNA translation initiation [18], whereas in case of the pSCodon plasmid we have not observed any adverse influence of the *lac* operator on the protein yield. This discrepancy could be explained by a insufficient spacing between the *lac* operator and the ribosome binding site in the pJP plasmid. The total yield of an unfolded bR was approximately 2 times lower than in the present study, because of a rapid degradation of the newly synthesized protein under the expression conditions.

Karnik et al. have shown that only 1–2% of bR synthesized in *E*. *coli* bound retinal, when cells were incubated at 37°C after induction with IPTG [18]. We suppose that the main factor that influences the yield of the functional bR in the case of *E*. *coli* expression may be the rate of protein synthesis. Indeed, it is recommended to express MPs at lower temperatures (20–30°C) to reduce the rate of the protein synthesis, facilitate its membrane insertion and proper folding [41]. As shown *in vitro* bR insertion into membrane occurs co-translationally in a Sec-dependent manner [37]. Its overexpression can overload the cell translocation system resulting in a misfolded protein and an increased rate of protein degradation. Induction of protein synthesis by lactose and cultivation of the cells at lowered temperature reduce the protein synthesis rate and thus favour an accumulation of the properly folded protein. Using similar mutations to optimize mRNA structure (-16.2 vs. -12.3 *kcal/mol*) we obtained the doubled translational yield of bR comparing to the previous study of Karnik *et al*. [18]. Since Karnik *et al*. employed a denaturing protein purification protocol, they had to renature the protein using DMPC/CHAPS vesicles, whereas our protocol takes advantage of non-denaturing conditions, thus allowing a straightforward production of the functional protein. The high rate of bR misfolding can also be attributed to an unfavorable lipid composition of the *E*. *coli* membrane. The PE lipids, a major component of the *E*. *coli* membrane, were shown to decrease the bR regeneration yield *in vitro* [42]. Also the folding of bR is described by a two-stage model with at least one transition state [43]. Therefore, *E*. *coli* lipids appear to have a significant effect on the transition state and favor bR misfolding.

Nevertheless, the protocol for high-yield bR production in *E*. *coli* introduced here allows one to obtain functional protein under non-denaturing conditions in quantities sufficient for structural biology and biochemical studies. Whereas other protocols require protein extraction with organic solvents and solubilization of the protein in denaturing detergents followed by protein renaturation as well as the necessity of the expression drivers [18–22]. bR obtained using the presented protocol is stable, homogenious and resembles a native bR from purple membranes, and thus satisfies the requirements for the use of this protein in different applications in science and industry.

One of the main advantages of the *E*. *coli* expression system over *H*. *salinarum* is the considerably reduced time required to produce the mutants of interest facilitating intense studies of the target protein. We proved that the suggested approach is also efficient for fast production of bR mutants. Asp85 is primary proton acceptor and Asp96 is primary proton donor, therefore the D85N and D96N are the key mutants that are intensively used to trap the intermediate states of bR. The higher expression level of D85N and D96N mutants compared to wild type bR deserves additional attention. It has been previously reported that mutation D94N in the bacteriorhodopsin from *Haloarcula marismortui* (analogous to D96N in bR) raised 10-fold the yield of the functional protein [44]. In addition, it was shown that insertion of the bR helix C into membrane *in vitro* is impeded by two aspartic acid residues within its transmembrane region [45]. In the present work D85N and D96N mutations led to the 1.5 to 4-fold increase in the yield of the functional protein relative to the wild type bR, the fraction of a properly folded protein increased as well from 25±10% to 35% and 60% of the total transcriptional yield of D85N and D96N mutants, respectively. This increase may be explained by improved incorporation of the newly synthesized protein into the *E*. *coli* membrane that reduces protein degradation and facilitates the correct folding of the bR mutants.

The growth of the first highly ordered 3D crystals of bR expressed in *E*. *coli* and the similarity of its structure to the structure of bR obtained from *H*. *salinarum* show the aptness of the presented approach for the expression of bR and its mutants suitable for all scientific and industrial applications. Moreover, the obtained high-quality crystals of bR produced in *E*. *coli* lacking native lipids of *H*. *salinarum* addresses a long-standing question in crystallization of membrane proteins. Despite the role of native lipids for MP crystallization being considered important, there is little information published on this matter [46]. Particularly, archea lipids that stabilize bR molecules in the trimer inside the 2D and 3D crystals were assumed to be highly specific [47, 48]. These findings were supporting the idea that native lipids of *H*. *salinarum* are required to form highly ordered bR crystals. Our work demonstrates that bR expressed in *E*. *coli* is readily crystallized by *in meso* approach and, therefore, native lipids surrounding the protein are not an absolute requirement for growing well diffracting bR crystals. Although we have demonstrated high efficiency of the CPA in the case of microbial rhodopsin family we believe that the CPA may be applied to other membrane proteins.

## Materials and Methods

### Materials

All the salts and media components were purchased either from AppliChem (Darmstadt, Germany) or Sigma-Aldrich (Taufkirchen, Germany) of analytical quality or higher. All enzymes were from Fermentas (part of Thermo Fisher Scientific, St. Leon-Rot, Germany). DDM was from Affymetrix (Santa Clara, USA), Sarkosyl from AppliChem, retinal, DMPC, and CHAPS from Sigma-Aldrich.

### DNA manipulations

The coding region of bR gene excluding the leader signal peptide sequence was amplified from pEF191 plasmid kindly provided by D. Oesterhelt [49]. The factor Xa cleavage site and eight histidine purification tag coding sequences were appended to the bR 3'-terminus using synthetic oligonucleotides resulting in GSGIEGRSGAPHHHHHHHH extension. The obtained bR gene was cloned into *Nde*I and *Xho*I sites of pSCodon vector (Delphi Genetics S.A., Charleroi, Belgium). The pSOPII plasmid bearing the SRII gene in pET27bmod vector was provided kindly by M. Engelhard [23]. All bR-SRII chimeric genes (Fig 2) and bR mutant genes were produced by PCR.

The mRNA folding energies were calculated using *mRNAshapes* software [31]. Bacteriorhodopsin coding sequence was optimized by introducing two silent mutations C9A and G12A corresponding to amino acids Ala2 and Gln3.

### Protein expression and membrane isolation

*E*. *coli SE1* [50] cells were transformed with pSCodon-derived plasmids, and *E*. *coli* BL21(DE3) cells were transformed with pET27-derived plasmids. Cells were plated over LB-agar and then grown in 450 ml rich ZYP-5052 autoinduction media [51] with an appropriate antibiotic in buffled shaking 2-L flasks at 120 rpm. The cells were incubated at 37°C until OD_600_ reached 1.0–1.2 AU, when 400 μl of 50 mM *all-trans* retinal solution in ethanol was added, and cells were further cultivated at 20°C. Cells from 1 litre of overnight culture were harvested by centrifugation at 5000 rpm, resuspended in 50 ml of 20 mM Tris-HCl pH 8.0, 5% glycerol, supplemented with 10 mg of lysozyme and 1 mg of DNAseI, and incubated for 2 h at 4°C with stirring. Lysate was obtained by passing the suspension 3 times through micro-fluidizer M-110P from Microfluidics (Westwood, USA). Then 5M NaCl was added to a final concentration of 200 mM and suspension was layered over a glycerol cushion (1 ml—90%, 1 ml—80%, 1ml—60%) in two 32 ml tubes. The total membranes were isolated by ultracentrifugation in SW-32Ti rotor (Beckman Coulter, Krefeld, Germany) at 28 000 rpm for 1h. The supernatant was discarded and the glycerol cushion containing membranes was resuspended in 50 ml of 20 mM Tris-HCl pH 8.0, 100 mM NaCl.

### Denaturing protein purification

Sarkosyl was added to the final concentration of 2% and membranes were solubilized overnight with stirring at 4°C. Insoluble material was removed by ultracentrifugation in Ti-70 (Beckman Coulter) rotor at 35 000 rpm for 1h. Supernatant was 5 times diluted with 20 mM Tris-HCl pH 8.0, 100 mM NaCl buffer and 10 mM imidazole was added. Suspension was loaded on the 5 ml of Ni-NTA resin (Qiagen, Hilden, Germany) equilibrated with the same buffer. The column was first washed with 3 CV of 50 mM Tris-HCl pH 8.0, 100 mM NaCl, 0.25% Sarkosyl, 20 mM imidazole buffer, then 5 CV of 100 mM Na_2_HPO_4_ pH 8.0, 100 mM NaCl, 0.2% SDS, and 3 CV of 100 mM Na_2_HPO_4_ pH 8.0, 0.2% SDS buffer. The protein was eluted with 3 CV of 100 mM Na_2_HPO_4_ pH 8.0, 0.2% SDS, 300 mM imidazole. To remove imidazole the samples were dialysed against 0.8 L of 50 mM NaH_2_PO_4_ pH 6.0, 0.2% SDS. When necessary purified proteins were refolded in mixed DMPC/CHAPS micelles [30]. Reliability of the protein yields comparison was estimated by Student's test and presented as *p* value.

### Non-denaturing protein purification

DDM was added to the final concentration of 1% and membranes were solubilized overnight with stirring at 4°C. Insoluble material was removed by ultracentrifugation in Ti-70 rotor at 35 000 rpm for 1h. Supernatant was 5 times diluted with 20 mM Tris-HCl pH 8.0, 100 mM NaCl buffer and 10 mM imidazole was added. Suspension was loaded on the 5 ml of Ni-NTA resin equilibrated with the same buffer. The column was washed with 10 CV of 50 mM NaH_2_PO_4_ pH 6.0, 100 mM NaCl, 0.2% DDM, 30 mM imidazole. The protein was eluted with 3 CV of 50 mM NaH_2_PO_4_ pH 7.4, 100mM NaCl, 0.2% DDM, 300 mM imidazole. Only coloured fractions were pooled. To remove imidazole the samples were immediately dialysed against 0.6 L of 50 mM NaH_2_PO_4_ pH 6.0, 100 mM NaCl for 2 hours and removed from dialysis buffer, then after 10 hours dialysis was continued for additional 2 hours against fresh buffer.

After the dialysis the protein heavily precipitated. The white pellet was separated by centrifugation at 5000 rpm from colored solution and discarded. Protein was concentrated to the volume of 2 ml by ultrafiltration and applied to 165 ml Sephacryl S200HR (GE Healthcare, Germany) column equilibrated with 50 mM NaH_2_PO_4_ pH 6.0, 100 mM NaCl, 0.1% DDM. Peak of colored functional protein could be easily separated from the peak of the aggregated protein.

The samples were protected from the light. Total protein concentration was measured by BCA Protein Assay Kit (Thermo Fisher Scientific, Schwerte, Germany) following supplier protocol. To access the protein purity the samples were analyzed on 8–16% gradient SDS-PAGE. UV-Vis absorbance spectrum was measured on UV-2450 spectrophotometer (Shimadzu, Duisburg, Germany). The fraction of the functional protein was accessed as absorbance ratio A_280_/A_λret_, where A_λret_ is maximum absorbance of retinal in the protein [52].

### Crystallization, data collection, and structure determination

The crystals were grown using *in meso* approach using nanovolume robotic system Formulatrix NT8 (Waltham, USA) [10, 33], similarly to our previous work [28, 53, 54]. The purified protein in crystallization buffer was added to the monooleoyl-based lipid mesophase. The best crystals were obtained using the protein concentration of 20 mg/ml and 1.5M Na/K-Pi pH 5.6 precipitation solution. The crystals were grown at 22°C and reached 120 μm in size in approximately one week.

X-ray diffraction data (wavelength 0.976 Å) were collected at the beamline ID23-1 of the European Synchrotron Radiation Facility (ESRF, Grenoble, France) using a PILATUS 6M detector (Dectris Ltd., Baden, Switzerland). Diffraction patterns integrated using XDS and the reflexes’ intensities were scaled using and XSCALE [55], and the space group was determined to be C2. The data statistics are presented in the Table 1. Initial phases were successfully obtained in the space group C2 by molecular replacement method using MOLREP [56], with PDB ID 1C3W [11] as a search model. There are three monomers in the asymmetric unit in this space group. The initial molecular replacement model was then iteratively refined using REFMAC5 [57] and Coot [58].

### Accession numbers

The atomic coordinate and structure factors have been deposited in the PDB with ID code 4XXJ.
